# AccessMod 3.0: computing geographic coverage and accessibility to health care services using anisotropic movement of patients

**DOI:** 10.1186/1476-072X-7-63

**Published:** 2008-12-16

**Authors:** Nicolas Ray, Steeve Ebener

**Affiliations:** 1 Computational and Molecular Population Genetics (CMPG), Institute of Ecology and Evolution, University of Bern, CH-3012 Bern, Switzerland; 2 Swiss Institute of Bioinformatics, Switzerland; 3 Information, Evidence and Research, World Health Organization, 20 av. Appia, 1211 Geneva 27, Switzerland

## Abstract

**Background:**

Access to health care can be described along four dimensions: geographic accessibility, availability, financial accessibility and acceptability. Geographic accessibility measures how physically accessible resources are for the population, while availability reflects what resources are available and in what amount. Combining these two types of measure into a single index provides a measure of geographic (or spatial) coverage, which is an important measure for assessing the degree of accessibility of a health care network.

**Results:**

This paper describes the latest version of AccessMod, an extension to the Geographical Information System ArcView 3.×, and provides an example of application of this tool. AccessMod 3 allows one to compute geographic coverage to health care using terrain information and population distribution. Four major types of analysis are available in AccessMod: (1) modeling the coverage of catchment areas linked to an existing health facility network based on travel time, to provide a measure of physical accessibility to health care; (2) modeling geographic coverage according to the availability of services; (3) projecting the coverage of a scaling-up of an existing network; (4) providing information for cost effectiveness analysis when little information about the existing network is available. In addition to integrating travelling time, population distribution and the population coverage capacity specific to each health facility in the network, AccessMod can incorporate the influence of landscape components (e.g. topography, river and road networks, vegetation) that impact travelling time to and from facilities. Topographical constraints can be taken into account through an anisotropic analysis that considers the direction of movement. We provide an example of the application of AccessMod in the southern part of Malawi that shows the influences of the landscape constraints and of the modes of transportation on geographic coverage.

**Conclusion:**

By incorporating the demand (population) and the supply (capacities of heath care centers), AccessMod provides a unifying tool to efficiently assess the geographic coverage of a network of health care facilities. This tool should be of particular interest to developing countries that have a relatively good geographic information on population distribution, terrain, and health facility locations.

## Background

In January 2003, Ministers of Health from seven countries (Chile, Germany, Greece, New Zealand, Slovenia, Sweden and the United Kingdom) established an international forum on common access to health care services [1]. This high level forum continues the emphasis on equitable access to health care established in the Constitution of the World Health Organization and the 1978 Declaration of Alma-Ata [2]. Despite longstanding initiatives, important gaps in access remain. For example, most countries will not meet the goal of universal access to antiretroviral treatment (ART) by 2010 [3].

In this paper, we use the conceptual framework of access to health care proposed by Peters *et al*. [4] that is derived from longstanding descriptions of access to health services [2,5,6]. This framework recognizes four dimensions of access: (1) geographic accessibility – the physical distance or travel time from service delivery point to the user; (2) availability – having the right type of care to those who need it; (3) financial accessibility – the relationship between the price of services and the willingness and ability of users to pay for those services; (4) acceptability – the match between how responsive health services providers are to the social and cultural expectations of individual users and communities. Central to this framework is the concept of the quality of care and each of these dimensions has a supply and demand concept.

Although financial accessibility and acceptability are important dimensions of access, we concentrate on geographic accessibility and availability in this paper. These two dimensions are certainly the less well understood [7], and in the context of health system performance, they can be converted into the measures of accessibility and availability coverage. Availability coverage reflects what resources are available and in what amount for delivering an intervention. This may include the number of health facilities, number of personnel, hours of operation, waiting time or the availability of different technologies (drugs, equipment, etc.). In other words, availability coverage relates the capacity of a health system to the size of the target population [8]. Accessibility coverage measures how physically accessible resources are for the population. The resources might be available but inconveniently located, therefore hindering physical access [8].

Independent analyses of availability and accessibility provide uni-dimensional perspectives: availability coverage describes how the supply of care is spatially distributed without considering if this supply is physically accessible, while accessibility coverage looks at how physically accessible a service is to the population without considering if the supply of care is sufficient to cover the demand. When availability and accessibility coverage are combined in a single analysis, one can define 'geographic (or spatial) coverage'. Analyzing geographic coverage requires taking into account conjointly the location and the maximum coverage capacity of each care provider, the geographic distribution of the population, the environment that the patient will have to cross to reach the care provider, as well as the transportation mode s/he will be using. Failing to account for the correct combination of these factors may greatly affect the geographic delineations of the catchment areas that are based on a maximum transportation time.

To efficiently address the spatially-explicit issues linked to geographic coverage one can benefit from Geographic Information Systems (GIS). GIS-based analysis is well established and has been applied in many areas including retail site analysis, transport, emergency service and health care planning [e.g. [9-11]]. In the context of health care planning, the ability of GIS to identify the geographic extent of a health facility catchment area, which corresponds to the area which contains the population potentially or actually utilizing this facility, is also a particularly important analytical capability [see e.g. [12]]. When most of the population is motorized (e.g. car, bus, motorcycle), the most common techniques involve a vector approach that relies on high quality road network information [13].

However, when people tend to use other types of transportation mode (e.g. walking or bicycling) or a combination of those with motorized transportation modes, raster GIS techniques are more commonly used because they do not restrict 'movement' only to the physical road network, but also incorporate travel across the 'terrain', which is particularly relevant in rural areas of many developing countries. The most common raster technique for analyzing movements across a continuous surface is the least-cost path approach [14,15] that calculates the least-cost distance between a focal location and all cells in the surroundings. This technique is found in most commercial GIS products such as the Spatial Analyst extension of ArcView and ArcGIS products (ESRI, Redlands, USA) or Idrisi (Clark Labs, Worcester, USA). In the context of accessibility to health care, the choice of an appropriate cost measure is a key decision, and very different results may be obtained depending on choice of measures [see [7,16]]. Two types of measures are generally considered: distance and time. There are several reasons for using travelling time rather than distance when measuring accessibility: (i) it is assumed that people more easily relate to travelling time rather than to geographic distance when making decisions on seeking care, (ii) travelling time is a more comparable measure (e.g. between countries) than distance because it can take transportation mode into account, (iii) level of care needed in an emergency is commonly measured in time.

Depending on the transportation mode, the travelling time to the 'nearest' health facility may be significantly influenced by the type of landcover and the presence of barriers to movement such as rivers or wetlands that the patient will have to cross or circumvent. Moreover, patient movements may be directionally dependent: the time taken to reach a health facility from a household is not necessarily equal to the time it takes for the return journey. This is referred to as 'anisotropy', and it may be due to topographical constraints that would increase travelling speeds when walking [17] or bicycling [18] down-slope. It has been demonstrated that the consideration of anisotropic movements may results in very different results than its 'isotropic' counterpart within the framework of cost-distance analysis of walking individuals [19].

Slopes are typically derived from a Digital Elevation Model (DEM). In the 'isotropic' method of the classical least-cost implementation, only the largest positive slope is considered between a focal grid cell and its eight neighboring cells, and the direction of movement has no effect on the implicit constraint. This is an appropriate method for watershed delineation where water flows through a topographical landscape, but is not realistic for the constraints imposed on moving individuals. The alternative 'anisotropic' approach considers slopes between any pairs of adjacent grid cells, and the direction of movement (upslope or downslope) becomes important to determine the constraint to movement.

AccessMod is a tool developed by WHO that uses the power of GIS to analyze physical accessibility and the geographic coverage of an existing health facility network in a mixed urban/rural context. It allows one to locate new health facilities in a scaling-up exercise. AccessMod can take into account the population distribution, the maximum capacity of each health facility in the network and travel scenarios which take into account different modes of transportation in an isotropic or anisotropic way to design the theoretical catchment area attached to each health facility. This paper describes the latest version of AccessMod (version 3.0), discusses the methodology underlying its capacities, presents an example application in the Southern part of Malawi and finally discusses the advantages as well as the limitations of the approach.

## Methods

### Technical considerations and data requirements

**Figure 1:**
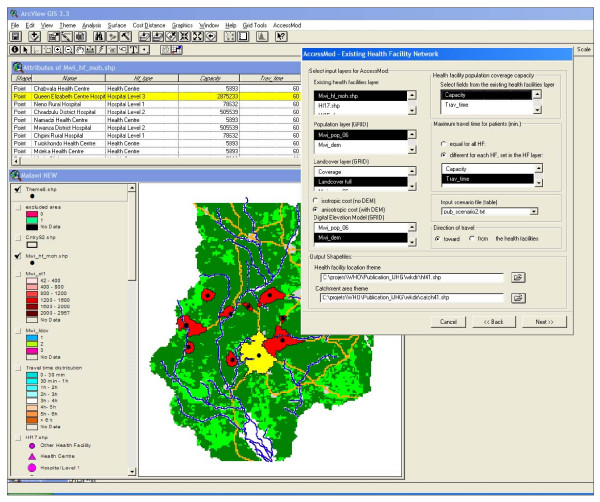
**Screenshot of an ArcView 3.×/AccessMod session**. The view shows outputs from the analysis of an existing network, as well as one of the dialog windows of AccessMod.

AccessMod is an extension to the Geographic Information System (GIS) ArcView 3.× (ESRI, Redlands, USA). The choice of the ArcView GIS platforms over others (e.g. IDRISI) was principally motivated by its widespread use and availability in developing countries, principally in Africa. Although ArcView 3.× is no longer maintained by ESRI, it has an extremely active user community. We plan to rewrite this extension for the ESRI ArcGIS platform, which should facilitate its use by a broader GIS community. AccessMod is a suite of graphical user interfaces facilitating interaction with the various types of analysis (see Figure 1) and the manipulation of graph structures (see details below). The modules are written in the Avenue language, and call two Windows dynamic linked libraries (dll) written in C++. The ArcView extension *Spatial Analyst *is required in order to manipulate grid layers. The AccessMod extension, along with a user and training guide and example data sets are available online from the WHO web site  and the script repository on the ESRI website .

AccessMod requires several data sets (see Table 1). Both raster and vector data are used as inputs, but the latter are always transformed into raster data during the analysis. Resolution issues may arise and the user must ensure that the input raster resolution is appropriate for the scale of the study. It is important that an equal-area projection is used for the data in order to avoid strong biases in the surface of the catchment areas and that meters are used as map units so that travelled distances are correctly linked to the user-defined travelling speeds expressed in km/h.

**Table 1:** Description of AccessMod input data sets. Names of mandatory data sets are in bold

**Type of data**	**Name**	**Description/additional information**
Raster	**population distribution grid**	Spatially-explicit distribution of population over the area. Point estimates coming from survey of administrative units should not be attributed to a single grid cell, but would need to be appropriately spread over the subunit surface of the administrative units

	**landuse grid**	Spatial distribution of the different categories of land use on which travelling speed may be different. This grid can be combined in AccessMod with additional landscape elements (e.g. roads, rivers) to obtain the final landcover grid

	digital elevation model (DEM)	Altitude distribution used to derive slopes and correct travelling speeds in the case of anisotropic movements

	exclusion area grid	In a scaling-up analysis, it can define an area where no new health facility can be placed but where some population might nevertheless be living (e.g. swamps, military zone, disaster prone areas, etc)

Vector	health facilities locations	This point shapefile contains the geographic locations of the existing network of health facilities. Its attribute table contains the population coverage capacity and the facultative maximum travelling time for each health facility

	road network	This line shapefile contains the road network. Different types of roads can be incorporated and combined with the landuse grid

	barriers to movements	Both line and polygon shapefiles can be treated as complete barriers to movement and can be integrated in the final landcover grid

Tabular	**travelling scenario**	This file defines the travelling speed and the mode of transportation (e.g. walking, bicycling) of each landcover

	new health facility information table	This file is used in the scaling-up analysis. It holds information on the different types of health facility and their associated population coverage capacity

### Overview of AccessMod capacities

AccessMod allows one to analyze the physical accessibility to health care using terrain information and population distribution. Four major modes of operations are available to the users. First, AccessMod can model the coverage of catchment areas linked to an existing health facility network based on travel time, measuring physical accessibility to health care. Second, it offers the capacity to measure the geographic coverage of the existing network by combining availability of care and physical accessibility into a single measure. Third, it implements a solution to complement an existing health facility network in the context of a scaling-up exercise. Finally, the scaling-up capacity can be used to provide information for cost effectiveness analysis when little information about the existing network is available.

AccessMod can seamlessly integrate travelling time, population distribution and the population coverage capacity specific to each health facility in the network. Moreover, various landscape components that can influence travelling time (e.g. topography, landuse type, road network, etc.) can readily be integrated into the various types of analysis. In version 3.0 of AccessMod, anisotropic movements of the patients due to topographical constraints are also taken into account. This means that the time taken to travel between a patient location (e.g. household) to the nearest health facility is not necessarily equal to the time it takes to do the same journey backwards.

### Computation of a theoretical catchment area

The notion of theoretical catchment area is central to the capacities of AccessMod. Given a health facility, its theoretical catchment area is defined as the surface from which patients are expected to be coming if financial accessibility and acceptability levels were equal among health facilities. Another strong assumption of the model is that patients always travel to the nearest health facility. Although there are specific field conditions where people may use informally trained health providers and shopkeepers, or by-pass nearby clinics in favor of farther ones [e.g. [20,21]], travelling to the nearest health facility may be considered as the correct behavior in the vast majority of situations.

The theoretical catchment is bounded in AccessMod by the combination of five factors:

1. the maximum travel time for patients to reach the health facility

2. the Health Facility Population Coverage Capacity (HFPCC)

3. the geographic distribution of the population

4. the landcover type(s) over which patient travelling occurs

5. a specific scenario regarding the travelling speed of various transportation modes used by the patient to reach the health facility

**Figure 2:**
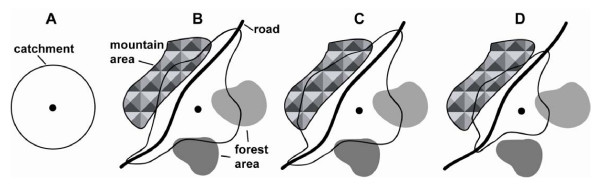
**Influence of landcover and population distribution on catchment area**. (A) circular catchment area centered on a health facility (black dot); (B) influence of land use and mode of transportation, (C) influence of anisotropic movement, with extended catchment in the mountain area; (D) influence of the distribution of the population. See text for details.

To facilitate the description of these factors and of the way they interact, we show in Figure 2 a sketch of the geographic extent of a hypothetical catchment area constraints by different combinations of these factors. We discuss below each of these factors and how they are taken into consideration in AccessMod.

Figure 2A shows the extent of a circular catchment. Assuming uniform landcover and topography, the catchment extension is only determined by a maximum travelling time which is reached in all directions at the same distance from the focal health facility. This maximum travelling time can be specific to each health facility and typically depends on several factors such as the type of services that are offered (e.g. emergency) or the severity of the patient's condition. For example, a specific emergency would require the patient to arrive within one hour to the health facility, while a planned operation typically gives more time for the patient to reach the same facility.

The first consideration in AccessMod is shown in Figure 2B, where different landcover (e.g. roads, forest, open bush) are taken into consideration and are attributed specific travelling speeds, but without any influence of the topography. This translates into a different shape of the catchment area. Zones with potential to be travelled through faster (e.g. roads) will have an extension of their catchment area, because more remote places can be reached within the given maximum travelling time. Technically, the computation of these catchment areas in AccessMod is done by the Dijkstra least-cost path algorithm [22] that relies on an underlying cost grid and a maximum accumulated cost [see [15] for details]. It assumes that the travelling time from any location to the health facility is always obtained by travelling along the optimum (i.e. fastest) route. This algorithm is the same as the one used in the function *costDistance *of ArcView 3.× and ArcGis, except that it can be used in AccessMod in anisotropic conditions (see below) and is computationally much faster. In our case, the cost given to each cell is the travelling time to cross the cell, which is determined through the travelling speed attributed to the landcover of the cell.

In Figure 2C, one can see the effect of considering anisotropic movements, where slopes are considered in all directions and where downhill walking is faster than walking uphill. Slopes are computed based on a digital elevation model (DEM). In anisotropic movements, the direction of travel is important. We assume here that we are interested in considering only patients moving toward the health facility, and their speed of travel would be therefore optimized on slopes that are downhill in the direction of the health facility. As a consequence of the anisotropy, the catchment area is modified and extents slightly further in the direction of higher topography.

Finally, Figure 2D shows how the population is taken into consideration. The extent of a catchment area should incorporate the supply of the health facility, which is its population coverage capacity. As the catchment area extents from the focal health facility, the algorithm sums up the population defined in an underlying population grid. As soon as the summed population reached the population coverage capacity, the algorithm stops, which delimits the extent of the catchment area (as is the case in Figure 2D). If the maximum travelling time is reached before the population coverage capacity, the final extent of the catchment area is only controlled by the travelling parameters and the health facility can therefore be considered as underserved.

### Integrating movements constraints and modes of transportation into a landcover grid

Analysing the physical accessibility to a health care network requires information on land uses, modes of transportation and travelling speed. AccessMod has been designed to facilitate the integration of all these pieces of information, and to optimize exploration of alternative scenario of transportation. A dedicated module in AccessMod allows the user to do the two steps involved in the development of a travelling scenario.

In a first step, a landcover grid can be assembled by combining a base land use grid with other landscape elements such as a road network, as well as linear and surface barriers to movement (see Table 1). The user can also choose whether roads have priorities when combined with barriers to movements. This choice is very important when, for example, a river network is considered as a barrier to movement. In that case, roads crossing some of the rivers segments are bridges that are not specifically described as such in the road network layer. Giving priorities to roads allows the river to be crossed on bridges and therefore permits the realistic extension of catchment areas. The combination of all landscape elements gives the full landcover grid that holds unique identifiers for each land use category.

**Figure 3:**
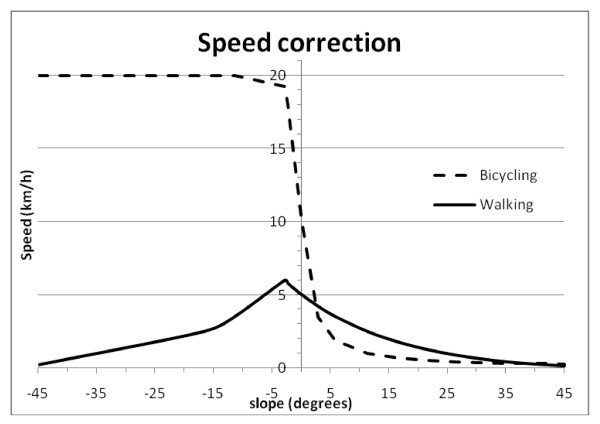
Speed correction for walking and bicycling depending on slope intensity.

In a second step, a travelling scenario must be set by the user. This scenario defines which of the landcover are used for patient travel, and what are the travelling speed and mean of transportation on each landcover. Each travelling speed (in km/h) can be set to any value by the user, but because travelling speeds are strong determinant of the realized extent of catchment areas, they should be chosen carefully based on known or supposed travelling habits of the population under consideration. Typically, relatively high speeds should be assigned to the road network, with different roads (e.g. secondary and primary roads, highways) having different travelling speeds. For other landcover types (e.g. forest, open bush), a mean walking speed on flat surface (e.g. 5 km/h) or mean bicycling speed on flat ground (e.g. 10 km/h) can be set. Two anisotropic speed correction models (using slopes derived for the DEM) can further be set by the user. A first model corrects for the walking speeds in hilly terrain and is derived from the Tobler's formula [17]. This correction basically decreases the effective speed of walking for up-slope and down-slope walking as the slope increases, while slightly increasing the effective speed for a slightly negative slope when walking down-slope. The second available model deals with speed correction for bicycling. We derived this correction using information on bicycle speed power calculation [18,23] and it assumes that the increased speed due to negative slope does not exceed twice the speed on flat ground. A graphical representation of the correction factors for walking and bicycling is shown in Figure 3.

Once a travelling scenario is defined, it can be used in any subsequent analysis that uses the landcover grid on which the scenario has been defined. This scenario can be easily modified in a text editor for testing of alternative travelling scenarios or for performing a sensitivity analysis of the travelling speeds attributed to the landcover.

Although AccessMod can handle different transportation modes with different speeds of travel, it is important to mention that the availability of the transport media is assumed to be similar over the analyzed area, which in reality is not necessarily the case. AccessMod does not currently include levels of mobility.

### Graph structure used in AccessMod

Among the different improvements which have been implemented in Accessmod 3 compared to the previous version 2.2 [see the complete list in ref. [24]], the use of an anisotropic Graph approach is the major one. We used the BOOST Graph Library (BGL) [25], a C++ open source library allowing one to construct any type of isotropic or anisotropic graph and providing many graph functions such as the Dijkstra least-cost path algorithm [22] utilized for the computation of catchment areas. Using the BGL, we developed the software MAPA (Mapping Anisotropy for Physical Accessibility), which can be compiled as a dynamic linked library (dll) for Windows. MAPA incorporates all the tools to compute the anisotropic traveling time map and to compute catchment areas. The MAPA dll is called by AccessMod 3.0 through the ARCVIEW scripting language *Avenue*.

**Figure 4:**
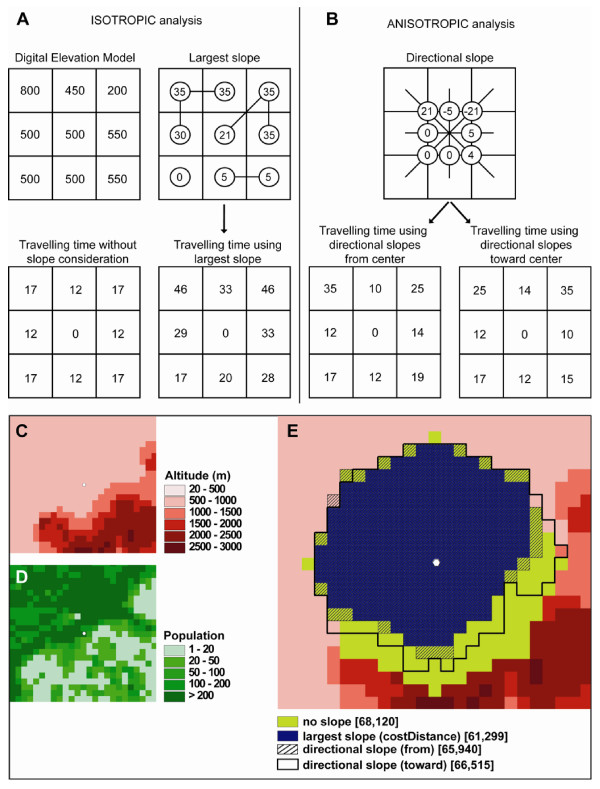
**Structure of isotropic and anisotropic graph in ArcView and AccessMod**. (A) Isotropic analysis case based on slopes derived from a DEM (values are in meters and cell width is 1 km). The largest slope between one cell and all its neighboring cells is attributed to the focal cell. The lines linking pairs of cells depict the direction of largest slope. The "travelling time without slope consideration" is obtained by considering a walking individual travelling at 5 km/h on flat ground. The "travelling time using largest slope" uses the largest slope values and correct travelling speeds through the Tobler formula (see text). (B) In the anisotropic analysis, slopes are computed between each cell and all its neighbors, and slope values are attributed to the arcs linking all pairs of cells. The directional slopes shown in the graph are computed from the center cell to its eight neighbors (using the same DEM than in (A)). The travelling times using directional slopes are derived using the Tobler formula, and can be either computed "from" the center cell or "toward" the center cell. (C) DEM used in the example; (D) population grid used in the example; (E) extent of a catchment area computed through four different ways of considering slopes. Numbers in brackets specify the population covered by each corresponding catchment.

In the classical *costDistance *function available in ArcView, each cell within the cost surface used as the input grid contains a single value representing the cost of movement across that location (cell) in any direction. It is a purely isotropic approach, with no consideration of the direction of movement. Moreover, when slopes are derived from a DEM and use as impediment to movement, only the largest slope value among the eight neighboring cells is kept and assigned to the focal cell (see Figure 4A). The graph structure that we have implemented in MAPA allows us no only to take into account the direction of movement in an anisotropic way, but also permits the computation and use of slopes in all directions to control how travelling time is computed between adjacent cells (see Figure 4B).

The few available tools using anisotropic functions compute least-cost paths *from *a given point, and not *in the direction *to this point. In our context, catchment areas of health facilities should be able to represent either inpatients or outpatients movements, and should therefore be able to consider travel from or toward the health facility. To achieve this, we use a property of anisotropic graphs: swapping the cost values of each pair of arcs between each pair of adjacent cells prior to computing the standard accumulative cost map from the health facility gives the catchment area toward this health facility. In AccessMod, the user can choose between these two modes of computation and can obtain complementary results for both in- and outpatients. As can be seen in a simple example in Figure 4B, the travelling time can be very different depending on the direction of travel. Because the travelling time directly impacts the extent of a catchment area, the served population can be significantly different when considering travel from or toward a health facility. This is exemplified in Figure 4E where we show extents of catchment areas, along with corresponding served population, for different ways of treating slopes in isotropic or anisotropic ways. We see in that Figure that failing to considering slope as an impediment to walking can lead to an overestimation of the served population. Considering slope in an isotropic way (only largest slope are considered) typically underestimates the served population, because only positive slopes are considered. The more realistic anisotropic way of considering slope give intermediate results that are nevertheless different between the two directional cases ("from" or "toward" the health facility).

### Analysis of an existing network of health facilities

Analyzing an existing network consists in evaluating its overall accessibility by the population of a given area. It is assumed that the populated area under consideration is "closed" in the sense that it cannot be served by health facilities located outside the area and that individuals from outside this area do not enter it in seek of care. This constraint is inherent to any surface-based spatial analysis applying a computation on all or part of an area. The analysis consists in looping over all health facilities and performing the following steps for each of them (details of the algorithm are described in the AccessMod user guide [24]):

1. computing its catchment area according to its population coverage capacity, the population grid, and the travelling constraints of the landscape (as shown in Figure 2);

2. clipping out the population served by the health facility (i.e. found underneath its catchment area) from the population grid;

3. moving to the next health facility using the updated population grid, and starting again at point 1 above.

A patient can be served by only one health facility. Moreover, the processing order of the health facilities may impact the results, and several alternatives are proposed by AccessMod. First, this order can be based on ascending (or descending) values of a field in the attribute table of the health facility data set (point shapefile). A typical field is the population coverage capacity, iterated in descending order, so that the largest health facilities are considered first in the analysis. Second, the number of people found in the proximity of each health facility can be computed and iterated upon. The computation can either be done using a circular buffer of a chosen radius or a catchment area of a given maximum travelling time.

Several outputs are produced after the analysis of an existing network:

- the attribute table of the shapefile of health facilities is updated with several variables such as the population found in the cell where the health facility is located, the total population located within the catchment area, and the travelling time observed at the limit of the catchment areas that have been designed (the maximum value here is the maximum travelling time set by the user);

- a polygon shapefile containing the extension of each catchment area;

- a grid containing the distribution of the population that could not be served by any health facility in the network. This unserved population grid can be used as the input population grid for a scaling-up analysis (see below);

- a grid portraying the coverage of the population in percent;

- a text file with several global statistics such as the percentage of population covered by the network, the percentage of total inhabited surface covered by the network, and the percentage of health facilities whose catchment areas extent to the maximum travelling time.

AccessMod also computes the strict 'physical accessibility' to the network. This analysis is only concerned by how travelling time is affected by landscape features, but it does not take the population into account. The output of this analysis is a map of travelling time to the nearest health facility. Although this type of analysis may be useful to get an idea, for example, of the required travelling time to a remote location, we do not recommend its use for measuring the coverage capacity of a health facility network because it does not incorporate the availability of care.

### Scaling up of an existing health facility network

The third feature of AccessMod projects the scaling up of an existing network by taking into account a population grid for which the population is assumed to be unserved by any existing health facility, and by determining the 'optimal' locations for additional health facilities. This capacity can also be used in the context of cost effectiveness analysis when little information about the existing network is available. In that case, the following methodology is simply applied on a complete population grid of a given area, in order to generate a completely new network of facilities covering all or part of the population in the area.

The first step in the analysis consists in targeting the most populated cell in the population grid, as it is considered to be more cost-effective to locate a health facility where the largest number of people is concentrated. Using a user-specified maximum travelling time, AccessMod then generates a first catchment area in order to determine which type of facility to implement at this location by looking at a health capacity table also provided by the user. This table defines the type(s) of health facilities that can be implemented, the corresponding minimum population coverage required for a cost-effective implementation, and the corresponding population coverage capacity.

Once the appropriate health facility type is selected, AccessMod uses the population coverage capacity and maximum travelling time to design the real catchment area following the same procedure than for analyzing an existing network. The maximum extension of the catchment area is obtained, results are stored in the outputs file and the analysis goes on to the next most populated cell in the grid and proceeds to the implementation of the second health facility. This iterative process continues until the specified number of facilities is reached. Details of the algorithm are described in the AccessMod user guide [24].

The main outputs of the scaling-up analysis are the following:

- a point and polygon shapefiles containing the locations of the new implemented health facilities and the extent of their corresponding catchment areas, respectively;

- a population grid containing the distribution of the population that could not be attributed to any health facility in the network. This grid can be used in a subsequent scaling-up analysis;

- a coverage grid that depicts the percentage of coverage of each cell in the grid.

## Application

**Figure 5:**
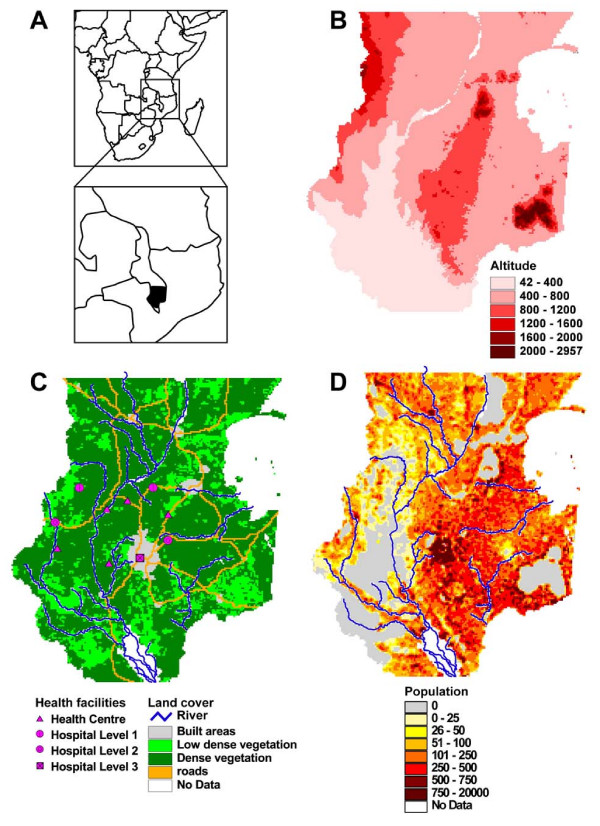
**Data sets used for the example analysis**. (A) Inset showing the location of Malawi and the area of interest in the southern part of the country; (B) Digital Elevation Model (DEM); (C) landcover grid with the river and road network, and the subset of 10 health facilities used in the analysis; (D) population grid with the river network. The southern wetland area (white polygon) is not considered in the analysis, and is treated as 'no data'.

To show the capacities of AccessMod under different realistic settings, we used data from the Southern part of Malawi (see Figure 5a). This area has been selected because it represents a mix of rural and urban areas, has different vegetation density levels, and several rivers form barriers to movements. The data sets used here are those that have been prepared for the AccessMod tutorial that is included in the download package:

• The 30 arc seconds Digital Elevation Model (DEM) called GTOPO30 and generated by the U.S. Geological Survey [26].

• The 1 km^2 ^population distribution grid created on the basis of the 1998 population census data, and provided by the National Statistical Office of Malawi. This data set has been adjusted to the 2006 census (Figure 5d). Cells located outside Malawi borders are assigned "No Data" values.

• A simplified version of the 1 km^2 ^Landcover grid generated from the 1998 Landscan Database [27] in which urban areas have been added from the Global Rural-Urban Mapping Project (GRUMP) data set [28]. In this grid, landcover is categorized into three classes (built area, low and dense vegetation).

• The geographic locations of a subset of 10 health facilities coming from a health facility census conducted by the Ministry of Health of Malawi in 2002–2003 [29]. In order to be used in AccessMod, the attribute table of this shapefile has been modified in order to contain the Population Coverage Capacity of each facility (Figure 1).

• The main roads network provided by the Survey Department of Malawi (Figure 5c). Some segments have been modified in order to cope for the resolution issue addressed in the discussion section of this paper.

• Part of the drain network (main rivers) (Figure 5c) and the Wetlands data set (Figure 5d) provided by the Survey Department of Malawi.

All these layers have been projected into the country specific UTM (zone 36) projection in order to have meters as map units. Note that we are only using a small subset of health facilities from the complete existing network, as well as only a part of the road network (main roads only). The results shown here are therefore only illustrative and must not be considered as reflecting the current level of health accessibility within this area (see the WHO/REACH TRUST/EQUINET/TARSC report [30] for an example of a complete assessment of access to HIV/AIDS care conducted in Malawi).

We define in Table 2 the four transportation scenarios that are being contrasted. These scenarios reflect real travelling situations in Malawi that have been informed through a patient exit survey conducted in Malawi in 2007 [30]. In scenario 1, all patients are walking to the nearest health facility. A walking speed is therefore attributed to each landcover type, with small speed differences depicting the influence of vegetation density. In scenario 2, it is assumed that patients first walk to the nearest main road or build area, then use a car to continue on their journey. Travelling speed are therefore much increased on main roads, and moderately so in build areas to reflect denser traffic. Scenario 3 is similar to scenario 2 but it considers that public transportation (bus) is used, and travelling speeds are thus decreased accordingly to reflect this slower transport media. In scenario 4, a bicycle is used on each landcover type, except in dense vegetation where it is supposed to be easier to walk and push one's bike alongside. For all scenarios, the DEM is used to compute slopes and assign anisotropic corrections to the walking and bicycling travelling speeds.

**Table 2:** Transportation scenarios

**Landcover type**	**Travelling speeds (km/h)**			
	**Scenario 1 all walking**	**Scenario 2 car + walking**	**Scenario 3 bus + walking**	**Scenario 4 bicycle + walking**

Build area	5 (W)	30	20	10 (B)

Low dense vegetation	4 (W)	4 (W)	4 (W)	7 (B)

Dense vegetation	3 (W)	3 (W)	3 (W)	3 (W)

Main road	5 (W)	80	50	10 (B)

(W): anisotropic correction for walking; (B): anisotropic correction for bicycling

**Figure 6:**
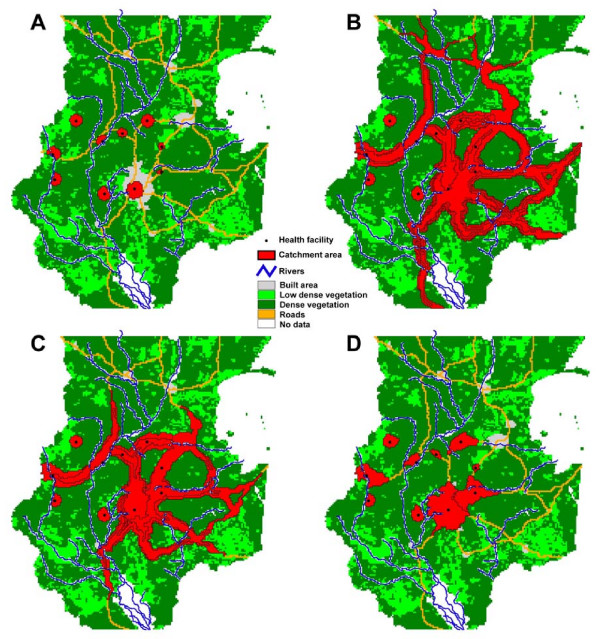
**Outputs of the analysis of the existing network of health facilities**. The extents of catchment areas are based on four travelling scenarios and a maximum travelling time of 90 minutes. (A) Only walking; (B) car + walking; (C) bus + walking; (D) bicycle + walking. Parameters of each scenario are defined in Table 2.

We show in Figure 6 the results of the analysis of the health facility network using each transportation scenario and a maximum travelling time of 90 minutes. For scenario 2 and 3, the catchments extend much further than for scenarios 1 and 4. This is due to the use of motor vehicle on roads that lengthen the travelling distance within the set maximum travelling time. Most of the catchments in scenario 1 are approximately circular, which reflects the uniformity of the landcover around the considered health facilities. However, some of these catchments have a truncated surface that is due to the intervening river network treated here as complete barrier to movement. This illustrates two important points regarding spatial data quality in AccessMod. First, the geographic coordinates of health facilities must be sufficiently accurate. A substantial shift in these coordinates can lead to a very different catchment area if, for example, this would place the health facility on the other side of a river or within a different type of landcover. This of course also applies to the other layers that are used in AccessMod (e.g. road and river networks, barriers to movements, extent of exclusion area).

Second, complete barriers to movement should be completed with appropriate crossing when it is effectively the case. Road bridges crossing main rivers are example of crossings that have important consequences in the analysis. Failure to account for these elements may lead to a subset of the population not being reached by a catchment area and to a potential underestimation of the covered population.

The covered population is also very different under each scenario presented in Figure 6. When considering scenario 2 and 3, 47.4% and 38.2% of the population is served, respectively. But only 8.5% and 20.3% of the population is served when considering the walking and bicycling scenario 1 and 4, respectively. For a given maximum travelling time, the mode of transportation in the southern part of Malawi has therefore a large impact on the served population. This calls for a careful consideration of how patients reach their nearest heath facility, and how the total population is distributed among the various transportation scenarios. With this type of information, AccessMod can be used to perform several analyses where each subpopulation is treated separately, and the results merged at the end by the user.

It is also interesting to contrast these results with results obtained with an isotropic approach (no consideration of slopes) and an anisotropic approach where travelling time is computed 'from' each of the health facilities. We show in Table 3 these alternative results. We can observed that the isotropic approach over-estimates the covered population, which is expected due to its unrealistic consideration of a complete flat 'landscape' with no impediments applied to travelling speeds. The results from the anisotropic approach computed 'from' the health facilities are slightly different from the corresponding ones computed 'toward' the health facilities, especially so for the scenario 1 and 4. The extent of the mismatch between the results of isotropic and anisotropic approaches depends on the topographical landscape, but it is expected that this mismatch be extreme in hilly or mountainous areas.

**Table 3:** Analysis results – existing network

**Type of analysis**	**% covered population**			
	**Scenario 1 all walking**	**Scenario 2 car + walking**	**Scenario 3 bus + walking**	**Scenario 4 bicycle + walking**

Isotropic analysis	9.7	51.3	41.7	19.8

Anisotropic analysis, travel toward HF	8.5	47.4	38.2	20.3

Anisotropic analysis, travel from HF	9.4	47.8	38.6	18.8

**Figure 7:**
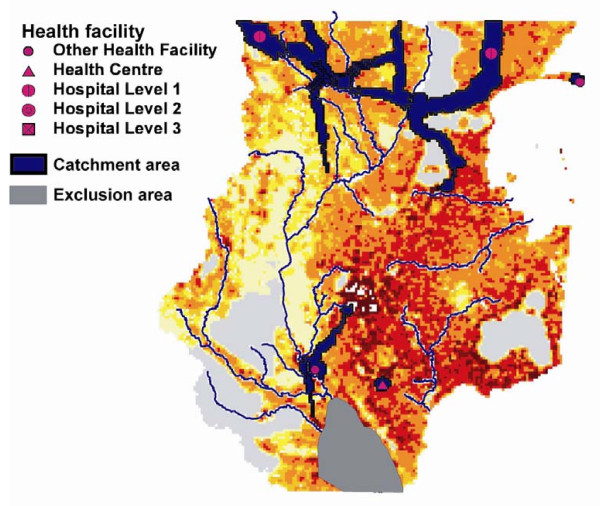
**Outputs of the scaling-up analysis**. Results show locations and corresponding catchment areas of five new health facilities based on a maximum travelling time of 90 minutes.

One of the results of the previous analysis is a population density grid showing the unserved population by the health facility network and the transportation scenario considered. This grid can be used in a subsequent scaling-up analysis, whose aim is to target optimized locations for new health facilities. In AccessMod, these optimized locations are cells with the largest unserved population, because it is considered to be more cost effective to prioritize these areas. We show in Figure 7 an example of such scaling-up exercise targeting five new health facilities with a maximum travelling time of 90 minutes. The base population density grid for this analysis was the one obtained after application of the analysis of the existing network under transportation scenario 2 (car + walking). Moreover, we considered an area unsuitable for new health facilities and that is located around the major southern wetland area. Note that AccessMod automatically integrates this unsuitable area in the analysis, but appropriately considers the population within this area to be reachable and accounted for by the catchment area of the new health facilities.

Results of the scaling-up analysis (Figure 7) show the five new health facilities located in targeted cells that have the highest population density, but that were not served by the existing network. The type of health facility for each location is different, depending both on the "new health facility information Table" (see Table 1) entered by the user and on the size of the population in each of the targeted cells. AccessMod gives priority to the type of health facility that would better serve the population in the targeted cell. This way of parametrizing the scaling-up analysis makes AccessMod very flexible to account for different types of resource allocation. For example, two scenarios could be contrasted in which a total budget is used to target several new small health facilities or one larger hospital. AccessMod could help contrasting the resulting served population under the two scenarios, and could therefore better inform the decision process.

## Discussion

Analyzing physical accessibility in an existing health facility network involves considering many factors that influence the time of travel. Moreover, the travelling time to the geographically nearest health facility is not sufficient to portray all aspects linked to access to health care. The availability (supply) of care provided by the health facility should also be taken into account. By integrating these two components through what is referred to as 'geographic coverage', AccessMod provides a more realistic analysis than alternative models which look at only one of these aspects (availability or geographic accessibility).

The approach using a travelling scenario with travelling speeds provided by the user and coupled to different modes of transportation (walking, driving, and bicycling) allows one to account for the many different field situations. However, it requires that data on modes of transportation, and especially on the percentage of the population using each mode, be available. This type of information may be obtained through appropriate surveys targeting patients linked to a subset of health facilities located in areas where different landcover dominate. When this information is lacking, it is important to carry out a sensitivity analysis on the maximum travelling time, the modes of transportation and the travelling speeds, in order to better understand which of these parameters is mostly responsible for variation in the output statistics of interest. The extent of catchment area is especially sensitive to the mode of transportation in areas that offer mixed landcovers with a developed road network.

The least-cost path algorithm assumption in the analysis of catchment area implies that travel always occur along optimum paths in term of total travelling time. The estimated travelling time is therefore assumed to be close to the travelling time perceived by patients and effectively realized. A few studies have addressed the accuracy of this assumption in developed countries and are based only on motorized travels [e.g. [31,32]]. They found that realized travelling time was close to the one modeled by the GIS. Nevertheless, one can assume that a small minority of travelling patients may be using other routes due to habits, social factors or other unknown parameters. This would be especially true when walking is the primary mode of transportation. However, the least-cost approach can be considered to reflect the overall tendency of travelling modes, and it serves as a useful mean statistical approach when a large area with many health facilities is analyzed, as advocated by others [e.g.[12]]. Thus, we strongly recommend this approach because it makes explicit the assumptions about travelling.

The consideration of anisotropic movements due to topographical constraints is based on physiological studies on individuals walking or bicycling. Moving up-slope or down-slope has a clear effect on the speed of movement and consequently on the extent of a catchment area (see Figure 7) and the total covered population (see Table 3). Using an anisotropic approach is meaningful in order to capture more realistically the population served by a network of health facility. The anisotropic effects will be enhanced in areas of rough topography, where individuals are necessarily travelling within rough terrain, without access to alternative flatter routes. It also appears that travelling scenarios involving bicycling are particularly affected by the way anisotropy is considered.

Another important advantage of AccessMod is the possibility of accounting for patient movement across borders. This is generally not considered when measuring population coverage because the underlying assumption is that each sub-national unit (e.g. province, district) is a closed system. This means that the population of the sub-unit is going to the health facilities located within the unit, and that these facilities only serve people coming from within this same unit. However, some considerations need to be taken into account in order to benefit from this advantage. If the analysis is to be performed on a unique sub-national unit, such as a district for example, a sufficient buffer, equivalent to twice the distance which can be covered at the maximum traveling speed, should be considered in order to account for overlapping catchment areas. If the analysis concerns a larger area within a country, such as the sample data set used here (Figure 5), we recommend applying AccessMod to the full country and then only consider the results obtained for the area of interest. The reason for this is exemplified in Figure 7, where the catchment areas of the three northern new health facilities are stopped by the boundaries of the considered area. Apart from insular territories, if the analysis needs to be applied on the complete surface of a country, the situation becomes more complex as the flow of patients through the country border might present completely different patterns than within the country itself. The ideal situation is again to use a buffer around the country under investigation, but this may not be possible due to the lack of appropriate spatial data sets for the neighboring countries.

Computing anisotropic movements imply setting the principal direction of movement from which the catchment area is defined. AccessMod can use either one of the two directions (from or toward the health facility) to derive the network of catchment area. However, it may be important in certain context to be able to account for the entire treatment time that comprises the travelling times toward and from the health facility, but additionally the time spent waiting and receiving the treatment at the health facility. In many cases, the waiting may be longer than the time taken by the patient traveling to and from the facility. By focusing only on the travelling time to or from the health facility, one may underestimate the overall time that the patient requires to receive care.

The use of a versatile graph structure in AccessMod permits the future incorporation of additional levels of realism into the computation of catchment areas. For example, it is possible to add a long distance rapid movement by adding an arc between two specific cells. This may represent transport of inpatients by plane or helicopter between two health centers, which cannot be readily incorporated into the standard input traveling time map. These special inpatient transports could be further linked to the level of treatment required (e.g. treated locally, treated by first level hospital), and different catchment areas could be obtained for each of these levels.

It is important to emphasize that AccessMod has mainly been developed to analyze a single type of service required. The assumption behind the health facility population coverage capacity (HFPCC) is that any health facility in the network can be chosen by the patient as long as this facility is reachable within the timeframe bounded by the maximum travelling time. This may not be realistic if the network of health facilities under study comprised very different services (e.g. emergency, non-emergency, and prevention-based services) that have very different attributed maximum travelling time. In such settings, it is important to do separate analysis by subdividing both the population grid and the network of health facilities in appropriate sets according to the type of service being investigated. A future version of AccessMod may facilitate this process by allowing the user to define how the population and the health facilities are allocated to different types of service.

When the population under consideration is the total population, another assumption of the model is that accessibility is gender neutral. This may not be the case in particular situations. For example, because of childbearing and child rearing, especially in high fertility settings, women are usually in more frequent contact with health facilities than men. Furthermore, as a reviewer noted, it is unusual for women to ride bicycles in Africa. However, the gender issue in access to health care is complex, and is likely to be more affected by the other aspects of accessibility than by its geographic component [see e.g. [33,34]]. As with the issue of the type of service, gender-specific analysis in AccessMod may be performed through distinct analyses in which the total population grid would have been separated in gender-specific sub-grids.

The spatial resolution of the analysis directly depends on the spatial resolution of the three grids that are used (DEM, population and landcover) and care should be taken when different options are available to the user. For example, using a relatively coarse resolution (e.g. 1–5 km), would imply that very local variations in slope would not be captured and that linear objects, such as roads or rivers, would be represented by objects much larger than they are in reality when integrated in the landcover distribution grid. This could results in geographically unrealistic features in the landscape such as artificial passages (bridge over a river for example). The final landuse should therefore always be checked thoroughly for these types of problems, and localized corrections may be required (e.g. when road segments fall into river segments). If this observation calls for the use of data at the highest resolution available, it should nevertheless be balanced with the amount of computer capacity available for the analysis as the memory requirement is linearly proportional to the number of grid cells over the study area [24]. Whenever several data resolutions are available, we recommend carrying out a sensitivity analysis to see the impact of alternative resolutions on the statistics to be derived from the analysis. Results of such a sensitivity analysis are likely to be extremely region- and data-specific, and can typically not be readily transferable from other studies. The number of countries for which the data are in sufficiently good quality and accuracy is still very limited. This shortage of adequate geographic information currently represents the major limitation towards the wide application of this type of approaches in developing countries.

The scaling-up module implemented in AccessMod may be useful in various processes such as health management, planning, operations, governance, financing, and policy. It can not only be used to compare alternative strategies based on different types of new health facilities, but can also show how overall geographic accessibility is leveraged with increasing number of new health facilities. The current limitation of this module is the way it gives priority to geographic locations where new health facilities can be implemented (i.e. in cells with highest population density). Other optimization could be imagined such as giving priority to areas that are far away from the existing network, that are close to the transport network, or a combination of those.

Financial accessibility and acceptability, the two other dimensions of accessibility to quality care [4], are not considered explicitly in the framework of AccessMod. This limitation may hinder the complete assessment of accessibility to care in a given region. However, information from studies specifically addressing these two dimensions exists in many countries [see [4], and references therein]. These studies could be used to segment the complete population grid into sub-grids reflecting the population found in different categories of financial accessibility and acceptability. AccessMod could then be run on these sub-grids, and results compared or combined.

In conclusion, AccessMod 3.0 represents a real improvement to both previous version of AccessMod and to other tools addressing accessibility to health care. These improvements make AccessMod a powerful tool for Ministries of Health to assess the geographic coverage of their existing health facility network and support scaling up when necessary. These capacities can not only be used for planning but also to determine other important issues such as inequities in access to care or population vulnerability to natural hazard for example [35]. Additionally, while developed in the context of access to health care, this extension can also be used to measure accessibility and geographic coverage for any other service or resource such as education, water, etc. We hope that the perspective of better informed decision making when analyzing accessibility and geographic coverage will lead to an improvement of the existing geographic information in countries. Because this geographic information is typically under the responsibility of different stakeholders (e.g. Survey Department, National Statistical Office, Ministry of Health, National Road Authority), it is also hoped that looking at accessibility to care, and this independently from the intervention being considered, could offer an additional powerful driver to support the development or the strengthening of the National Spatial Data Infrastructure (NSDI) process in these countries.

## Competing interests

The authors declare that they have no competing interests.

## Authors' contributions

NR programmed the extension and the associated DLLs, and did the analysis and Figures. SE supervised the project. NR and SE developed the algorithms and methods, and wrote the paper.

## References

[B1] Oliver A, Mossialos E (2004). Equity of access to health care: outlining the foundations for action. Journal of Epidemiology and Community Health.

[B2] WHO (1978). Declaration of Alma-Ata. International Conference on Primary Health Care: 1978; Alma-Ata, USSR.

[B3] WHO/UNAIDS/UNICEF (2008). Towards Universal Access Scaling up priority HIV/AIDS interventions in the health sector – Progress Report.

[B4] Peters DH, Garg A, Bloom G, Walker DG, Brieger WR, Rahman MH (2008). Poverty and Access to Health Care in Developing Countries. Annals of the New York Academy of Sciences.

[B5] Aday LA, Andersen R (1974). A Framework for the Study of Access to Medical Care. Health Serv Res.

[B6] Penchansky R, Thomas JW (1981). The concept of access: definition and relationship to consumer satisfaction. Med Care.

[B7] Guagliardo M (2004). Spatial accessibility of primary care: concepts, methods and challenges. International Journal of Health Geographics.

[B8] Tanahashi T (1978). Health service coverage and its evaluation. Bulletin of the World Health Organization.

[B9] Wilkinson P, Grundy C, Landon M, Stevenson S, Gatrell A, Loytonen M (1998). GIS in public health. GIS and Health.

[B10] Cromley EK, McLafferty SL (2002). Analyzing Access to Health Services. GIS and Public Health.

[B11] Albert DP, Gesler WM, Horner RD, Albert DP, Gesler WM, Levergood B (2000). Geographic Information Systems in Health Services Research. Spatial Analysis, GIS and Remote Sensing Applications in the Health Sciences.

[B12] Brabyn L, Skelly C (2002). Modeling population access to New Zealand public hospitals. International Journal of Health Geographics.

[B13] Bagheri N, Benwell G, Holt A (2005). Measuring spatial accessibility to primary health care. The 17th Annual Colloquium of the Spatial Information Research Centre.

[B14] Longley PA, Goodchild MF, Maguire DJ, Rhind DW (2005). Geographical Information Systems & Science.

[B15] Adriaensen F, Chardon JP, De Blust G, Swinnen E, Villalba S, Gulinck H, Matthysen E (2003). The application of 'least-cost' modelling as a functional landscape model. Landscape and Urban Planning.

[B16] Apparicio P, Abdelmajid M, Riva M, Shearmur R (2008). Comparing alternative approaches to measuring the geographical accessibility of urban health services: Distance types and aggregation-error issues. International Journal of Health Geographics.

[B17] Tobler W (1993). Three presentations on geographical analysis and modeling: National Center for Geographic Information and Analysis, University of California, Santa Barbara. Technical Report 93-1.

[B18] Bicycle power calculation. http://www.kreuzotter.de/english/espeed.htm.

[B19] Wood BM, Wood ZJ (2006). Energetically optimal travel across terrain: visualizations and a new metric of geographic distance with archaeological applications. SPIE Electronic Imaging: 2006; San Jose.

[B20] Leonard KL, Mliga GR, Haile Mariam D (2002). Bypassing Health Centres in Tanzania: Revealed Preferences for Quality. J Afr Econ.

[B21] Akin J, Hutchinson P (1999). Health-care facility choice and the phenomenon of bypassing. Health Policy Plan.

[B22] Dijkstra EW (1959). A note on two problems in connect with graphs. Numerishe Mathematik.

[B23] Bicycle Velocity Prediction. http://bikecalculator.com/.

[B24] Ray N, Ebener S (2008). ACCESSMOD3. Physical Accessibility to Health Care and Population coverage Modeling – User Manual. WHO, Geneva.

[B25] Siek JG, Lee L, Lumsdaine A (2001). The Boost Graph Library: User Guide and Reference Manual. Addison Wesley Professional.

[B26] GTOPO30: a global digital elevation model at 30 arc-second resolution. http://edc.usgs.gov/products/elevation/gtopo30.html.

[B27] LandScanTM Global Population Database. http://www.ornl.gov/landscan.

[B28] Global Rural-Urban Mapping Project (GRUMP): Gridded population of the world. http://sedac.ciesin.columbia.edu/gpw.

[B29] National health facilities inventory survey in Malawi, health facilities database, Malawi (2002–2003).

[B30] (2007). WHO and REACH TRUST/EQUINET/TARSC. Guidelines for indicators for monitoring equity and health systems strengthening in access to antiretroviral therapy – Promoting Equity and a health systems approach towards treatment access and responses to HIV and AIDS in Southern Africa. Guidelines to monitoring equity in access to ART.

[B31] Haynes R, Jones A, Sauerzapf V, Zhao H (2006). Validation of travel times to hospital estimated by GIS. International Journal of Health Geographics.

[B32] Fone D, Christie S, Lester N (2006). Comparison of perceived and modelled geographical access to accident and emergency departments: a cross-sectional analysis from the Caerphilly Health and Social Needs Study. International Journal of Health Geographics.

[B33] Ulasi I (2008). Gender bias in access to healthcare in Nigeria: a study of end-stage renal disease. Tropical Doctor.

[B34] International Community of Women Living with HIV/AIDS (ICW) and the Global Coalition on Women and AIDS (GCWA). Fact sheet – Access to care, treatment and support (ACTS). http://www.icw.org/files/ACTS-ICW%20fact%20sheet-06_0.doc.

[B35] El Morjani ZEA, Ebener S, Boos J, Abdel Ghaffar E, Musani A (2007). Modelling the spatial distribution of five natural hazards in the context of the WHO/EMRO Atlas of Disaster Risk as a step towards the reduction of the health impact related to disasters. International Journal of Health Geographics.

